# Developmental Profiles of Eczema, Wheeze, and Rhinitis: Two Population-Based Birth Cohort Studies

**DOI:** 10.1371/journal.pmed.1001748

**Published:** 2014-10-21

**Authors:** Danielle C. M. Belgrave, Raquel Granell, Angela Simpson, John Guiver, Christopher Bishop, Iain Buchan, A. John Henderson, Adnan Custovic

**Affiliations:** 1 Centre for Respiratory Medicine and Allergy, Institute of Inflammation and Repair, University of Manchester and University Hospital of South Manchester, Manchester, United Kingdom; 2 Centre for Health Informatics, Institute of Population Health, University of Manchester, Manchester, United Kingdom; 3 School of Social and Community Medicine, University of Bristol, Bristol, United Kingdom; 4 Microsoft Research Cambridge, Cambridge, United Kingdom; Simon Fraser University, Canada

## Abstract

**Background:**

The term “atopic march” has been used to imply a natural progression of a cascade of symptoms from eczema to asthma and rhinitis through childhood. We hypothesize that this expression does not adequately describe the natural history of eczema, wheeze, and rhinitis during childhood. We propose that this paradigm arose from cross-sectional analyses of longitudinal studies, and may reflect a population pattern that may not predominate at the individual level.

**Methods and Findings:**

Data from 9,801 children in two population-based birth cohorts were used to determine individual profiles of eczema, wheeze, and rhinitis and whether the manifestations of these symptoms followed an atopic march pattern. Children were assessed at ages 1, 3, 5, 8, and 11 y. We used Bayesian machine learning methods to identify distinct latent classes based on individual profiles of eczema, wheeze, and rhinitis. This approach allowed us to identify groups of children with similar patterns of eczema, wheeze, and rhinitis over time.

Using a latent disease profile model, the data were best described by eight latent classes: no disease (51.3%), atopic march (3.1%), persistent eczema and wheeze (2.7%), persistent eczema with later-onset rhinitis (4.7%), persistent wheeze with later-onset rhinitis (5.7%), transient wheeze (7.7%), eczema only (15.3%), and rhinitis only (9.6%). When latent variable modelling was carried out separately for the two cohorts, similar results were obtained. Highly concordant patterns of sensitisation were associated with different profiles of eczema, rhinitis, and wheeze. The main limitation of this study was the difference in wording of the questions used to ascertain the presence of eczema, wheeze, and rhinitis in the two cohorts.

**Conclusions:**

The developmental profiles of eczema, wheeze, and rhinitis are heterogeneous; only a small proportion of children (∼7% of those with symptoms) follow trajectory profiles resembling the atopic march.

*Please see later in the article for the Editors' Summary*

## Abstract 2

Using data from two population-based birth cohorts, Danielle Belgrave and colleagues examine the evidence for atopic march in developmental profiles for allergic disorders.

*Please see later in the article for the Editors' Summary*

## Editors' Summary

**Background:**

Our immune system protects us from viruses, bacteria, and other pathogens by recognizing specific molecules on the invader's surface and initiating a sequence of events that culminates in the death of the pathogen. Sometimes, however, our immune system responds to harmless materials (allergens such as pollen) and triggers allergic, or atopic, symptoms. Common atopic symptoms include eczema (transient dry itchy patches on the skin), wheeze (high pitched whistling in the chest, a symptom of asthma), and rhinitis (sneezing or a runny nose in the absence of a cold or influenza). All these symptoms are very common during childhood, but recent epidemiological studies (examinations of the patterns and causes of diseases in a population) have revealed age-related changes in the proportions of children affected by each symptom. So, for example, eczema is more common in infants than in school-age children. These findings have led to the idea of “atopic march,” a natural progression of symptoms within individual children that starts with eczema, then progresses to wheeze and finally rhinitis.

**Why Was This Study Done?:**

The concept of atopic march has led to the initiation of studies that aim to prevent the development of asthma in children who are thought to be at risk of asthma because they have eczema. Moreover, some guidelines recommend that clinicians tell parents that children with eczema may later develop asthma or rhinitis. However, because of the design of the epidemiological studies that support the concept of atopic march, children with eczema who later develop wheeze and rhinitis may actually belong to a distinct subgroup of children, rather than representing the typical progression of atopic diseases. It is important to know whether atopic march adequately describes the natural history of atopic diseases during childhood to avoid the imposition of unnecessary strategies on children with eczema to prevent asthma. Here, the researchers use machine learning techniques to model the developmental profiles of eczema, wheeze, and rhinitis during childhood in two large population-based birth cohorts by taking into account time-related (longitudinal) changes in symptoms within individuals. Machine learning is a data-driven approach that identifies structure within the data (for example, a typical progression of symptoms) using unsupervised learning of latent variables (variables that are not directly measured but are inferred from other observable characteristics).

**What Did the Researchers Do and Find?:**

The researchers used data from two UK birth cohorts—the Avon Longitudinal Study of Parents and Children (ALSPAC) and the Manchester Asthma and Allergy Study (MAAS)—for their study (9,801 children in total). Both studies enrolled children at birth and monitored their subsequent health at regular review clinics. At each review clinic, information about eczema, wheeze, and rhinitis was collected from the parents using validated questionnaires. The researchers then used these data and machine learning methods to identify groups of children with similar patterns of onset of eczema, wheeze, and rhinitis over the first 11 years of life. Using a type of statistical model called a latent disease profile model, the researchers found that the data were best described by eight latent classes—no disease (51.3% of the children), atopic march (3.1%), persistent eczema and wheeze (2.7%), persistent eczema with later-onset rhinitis (4.7%), persistent wheeze with later-onset rhinitis (5.7%), transient wheeze (7.7%), eczema only (15.3%), and rhinitis only (9.6%).

**What Do These Findings Mean?:**

These findings show that, in two large UK birth cohorts, the developmental profiles of eczema, wheeze, and rhinitis were heterogeneous. Most notably, the progression of symptoms fitted the profile of atopic march in fewer than 7% of children with symptoms. The researchers acknowledge that their study has some limitations. For example, small differences in the wording of the questions used to gather information from parents about their children's symptoms in the two cohorts may have slightly affected the findings. However, based on their findings, the researchers propose that, because eczema, wheeze, and rhinitis are common, these symptoms often coexist in individuals, but as independent entities rather than as a linked progression of symptoms. Thus, using eczema as an indicator of subsequent asthma risk and assigning “preventative” measures to children with eczema is flawed. Importantly, clinicians need to understand the heterogeneity of patterns of atopic diseases in children and to communicate this variability to parents when advising them about the development and resolution of atopic symptoms in their children.

**Additional Information:**

Please access these websites via the online version of this summary at http://dx.doi.org/10.1371/journal.pmed.1001748.

- The UK National Health Service Choices website provides information about eczema (including personal stories), asthma (including personal stories), and rhinitis
- The US National Institute of Allergy and Infectious Diseases provides information about atopic diseases
- The UK not-for-profit organization Allergy UK provides information about atopic diseases and a description of the atopic march
- MedlinePlus encyclopedia has pages on eczema, wheezing, and rhinitis (in English and Spanish)
- MedlinePlus provides links to further resources about allergies, eczema, and asthma (in English and Spanish)
- Information about ALSPAC and MAAS is available
- Wikipedia has pages on machine learning and latent disease profile models (note that Wikipedia is a free online encyclopedia that anyone can edit; available in several languages)

## Introduction

In the last two decades, the term “atopic march” has been widely used to describe temporal changes in the prevalence of childhood eczema, asthma, and allergic rhinitis reported in epidemiological studies [1]–[4]. These studies led to the hypothesis that the changes observed at the population level were a consequence of the progression of a cascade of symptoms within individual patients, starting with eczema, progressing to asthma, and then to rhinitis (with a resolution of some of the early eczema with increasing age) [1]–[3],[5]–[9]. It has been proposed that the observed temporal relationship between atopic diseases may help earlier diagnosis, and may facilitate novel approaches to disease prevention [10]. Practical clinical guidelines in both the US [11] and the UK [12] refer to the existence of the atopic march, and the leading UK guideline recommends that healthcare professionals inform parents that children with atopic eczema can often develop asthma and/or allergic rhinitis. Indeed, this conceptual framework has been used to design studies aiming to prevent the development of airway inflammation and asthma in children who were deemed to be at risk by virtue of having eczema and a family history of atopic disease [13]. However, despite considerable effort and investments in such studies, there is no evidence that targeting children with eczema with an intervention designed to modify the progression towards asthma is effective [14]. Of note, if atopic march does not accurately describe the developmental profiles of eczema, asthma, and rhinitis at an individual level, then stratifying children in this way to asthma prevention programmes may not be appropriate.

Most of the studies that associated early-life eczema with an increased risk of subsequent asthma and allergic rhinitis used cross-sectional data analyses (even within longitudinal studies), or longitudinal analyses based on few time points, without taking into account the time course of the development of symptoms within individual children's longitudinal profiles [2],[5],[15]–[21]. The existence of the atopic march has been questioned [22]–[24], with some studies suggesting that there is heterogeneity between patients in the chronology of the development of symptoms [25]–[27]. Children with early eczema who later develop asthma and rhinitis may belong to a distinct phenotype, rather than representing the typical progression of atopic diseases [27]. A recent review concluded that despite apparent clear temporal association linking eczema with progression to asthma and allergic rhinitis (and plausible biological mechanisms to underpin such associations), there is no definitive proof that atopic march is causal [4].

The emphasis on atopic march without robust evidence to support this notion may hamper research into the underpinning pathophysiology of atopy-related diseases. We propose that eczema, wheeze, and rhinitis may appear to follow an atopic march pattern if the prevalence or incidence of disease is investigated cross-sectionally at a population level, but that this may not best describe the trajectory of disease within most individual patients. Recent developments in machine learning provide new ways to capture the heterogeneity in longitudinal patterns of distinct symptom categories within individual patients, where conventional modelling techniques might over-aggregate the underlying complexity [28]. Machine learning is a data-driven approach to identify structure within the data using unsupervised learning of latent variables. It is used commonly by computer scientists for problem-solving in many other fields, and is increasingly used to disaggregate complex disease phenotypes in respiratory medicine and allergy [29]–[32]. In machine learning, latent variable modelling allows us to hypothesize the existence of an underlying trait that is not directly observed or measured, but whose presence can be indirectly inferred by the existence of different observed patterns of symptoms. A latent variable is a hypothetical construct used to describe generalised manifestations that cannot be directly measured, but whose presence may be inferred based on a set of observable characteristics or features (e.g., similar patterns of responses to a series of questions). These variables are widely used in the psychiatric literature to classify varying severity of constructs, such as “intelligence”, “depression”, and “autism”, that cannot be directly measured [33],[34] or discrete latent classes or categories of latent variables such as “severe depression”, “mild depression”, and “intermediate depression”. Within the context of the current study, machine learning may facilitate better understanding of how the relationships between symptom transitions can be generalised to identify patterns of symptoms within children. We hypothesize that the atopic march is an imposed paradigm that does not adequately describe the natural history of eczema, wheeze, and rhinitis during childhood, and that a data-driven approach may help us to identify developmental profiles of symptoms over time by uncovering the latent structure in the data. To address this hypothesis, we used machine learning techniques to model the development of eczema, wheeze, and rhinitis during childhood in two large population-based birth cohorts, taking into account longitudinal changes within individual children.

## Methods

### Design, Setting, Participants, and Data Sources

We studied two population-based birth cohorts from the UK: the Avon Longitudinal Study of Parents and Children (ALSPAC) [35] and the Manchester Asthma and Allergy Study (MAAS) [36]. Both studies were approved by local research ethics committees. Written informed consent was obtained from all parents. Participants were recruited prenatally, and followed prospectively. We administered validated questionnaires to collect information on parentally reported symptoms at review clinics at five comparable follow-up points (ages 1, 3, 5, 8, and 11 y). We included children who had data available for at least two time points for each symptom.

#### Manchester Asthma and Allergy Study

MAAS was a population-based birth cohort study that included the maternity catchment area of Wythenshawe and Stepping Hill Hospitals (50 square miles of South Manchester and Cheshire), a stable mixed urban-rural population. All pregnant women were screened for eligibility at antenatal visits (8th–10th week of pregnancy) between 1 October 1995 and 1 July 1997. Of the 1,499 women and their partners who met the inclusion criteria (<10 wk of pregnancy, maternal age>18 y, questionnaire and skin test data available for both parents), 288 declined to take part in the study, and 27 were lost to follow-up between recruitment and childbirth. A total of 1,184 participants had at least some evaluable data, and 1,136 participants had data for at least two time points.

Children attended review clinics at ages 1, 3, 5, 8, and 11 y. Validated American Thoracic Society–Division of Lung Disease (ATS-DLD-78) [37] (age 1 and 3 y) and International Study of Asthma and Allergies in Childhood (ISAAC) [38] (age 5, 8, and 11 y) questionnaires were interviewer-administered to determine parentally reported history of wheeze, eczema, and rhinitis, and treatments received. Atopic sensitisation was ascertained by skin prick testing for common inhalant and food allergens [29]. We also extracted all data from electronic and paper-based primary care medical records, including topical corticosteroid prescriptions for eczema.

#### Avon Longitudinal Study of Parents and Children

ALSPAC was a population-based birth cohort study in Avon, United Kingdom. ALSPAC was additionally approved by the ALSPAC Law and Ethics Committee. ALSPAC recruited 14,541 pregnant women between 1 April 1991 and 31 December 1992. A total of 8,665 participants had data for at least two time points.

Children attended review clinics at ages 1.5, 3.5, 4.5, 8.6, and 10.7 y. A modified ISAAC questionnaire [38] was administered to determine parentally reported history of wheeze, eczema, and rhinitis and treatments received. Skin prick testing was carried out at the follow-up at age 8.6 y.

### Definition of Variables

#### Current eczema

This variable was defined as a positive answer to the question “Has your child had an itchy rash that comes and goes in the last 12 months?” [38] (ALSPAC, all time points; MAAS, ages 5, 8, and 11 y) or “Has your doctor ever told you that your child has eczema?” [37] (MAAS, age 1 and 3 y).

#### Current wheeze

This variable was defined as a positive answer to the question “In the last 12 months has he/she had any periods when there was wheezing or wheezing with whistling on his/her chest when he/she breathed?” (ALSPAC) or “Has your child had wheezing or whistling in the chest in the last 12 months?” (MAAS).

#### Current rhinitis

This variable was defined as a positive answer to the question “In the past 12 months, has your child had a problem with sneezing or a runny or blocked nose when he/she did not have a cold or the flu?” [38] (ALSPAC and MAAS, ages 5, 8, and 11 y) or “Has your doctor ever told you that your child has hay fever or allergic rhinitis?” [37] (MAAS, age 1 and 3 y).

#### Current asthma

This variable was defined as the presence of any two of the following three features: current wheeze, current use of asthma medication, or physician-diagnosed asthma ever.

#### Atopic sensitisation

We defined sensitisation as a wheal diameter 3 mm greater than the negative control to at least one allergen.

#### Moderate/severe eczema

This variable was defined in MAAS as parentally reported eczema (questionnaires) *and* confirmed topical corticosteroid prescriptions for eczema by primary care physician (medical records).

### Statistical Analysis

Longitudinal data were jointly modelled across the two cohorts, and separately for each cohort. We used a Bayesian machine learning modelling framework [28] with an expectation propagation algorithm [39] for approximate inference to identify distinct latent classes based on individual changing disease profiles of eczema, wheeze, and rhinitis. We hypothesized that these different patterns represent the existence of distinct latent classes. All machine learning models were specified using Infer.NET (http://research.microsoft.com/infernet) [40].

In order to capture disease heterogeneity and encapsulate possible different patterns of symptom progression in individual children over time, we investigated three models. We first used two Markov chain probability models that assumed that the probability of an event depends on the disease state at the previous time point. These models allowed us to test the assumption that transitions or the sequence from one disease state to another follow the atopic march profile. For each of the models, we assumed a discrete time model at time steps where data were available (i.e., at ages 1, 3, 5, 8, and 11 y).

#### Model 1

**Figure 1:**
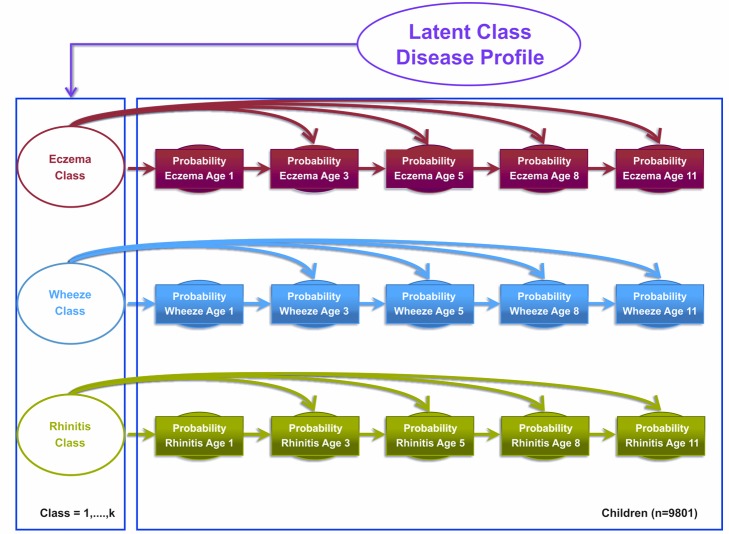
Graphical representation of the independent Markov chain model where transitions within each symptom are assumed to be independent (Model 1). Shaded circles represent observed variables, and unshaded circles represent latent variables to be inferred. Symptoms are joined together by a latent class disease profile.

Model 1 assumed that eczema, wheeze, and rhinitis profiles follow independent disease profiles over time that are linked by a single multinomial latent variable. This model assumed that the transition of acquiring, retaining, or remitting each disease followed an independent Markov chain of events, i.e., the probability of each disease depends on the previous state of that particular disease. In other words, the probability of having (or not having) eczema at time *t* is conditional on the probability of eczema at time *t*−1, which in turn is conditional on the probability of eczema at time *t*−2, with identical transition probability assumptions for the probability of wheeze and rhinitis. These three independent transition probability chains were governed by three latent disease classes, represented as an eczema class, a wheeze class, and a rhinitis class. Binary responses are linked via a probit link function. Figure 1 summarises the model specification.

#### Model 2

**Figure 2:**
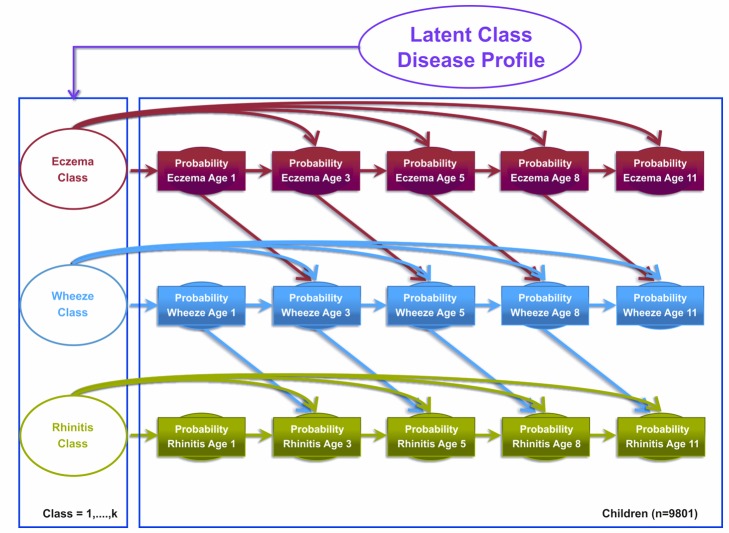
Graphical representation of the Markov chain model allowing transition probabilities across symptoms to follow an atopic march progression of symptoms (Model 2). Shaded circles represent observed variables, and unshaded circles represent latent variables to be inferred. Symptoms are joined together by a latent class disease profile.

Model 2 was the atopic march model, which assumed that the transition states proposed by the atopic march give a more optimal description of the data. This model assumed explicit probability transitions of progression from one disease to another (e.g., eczema at time *t* predicts wheeze at time *t*+1; Figure 2). This model allows eczema at time *t*−1 to condition the probability of wheeze at time *t*, and wheeze at time *t* to condition the probability of rhinitis at time *t*+1. These transitions resemble the progression hypothesized by the atopic march of symptoms, where eczema predicts later predisposition to wheeze, which in turn predicts later onset of rhinitis. Patterns of transition from one disease state to another were governed by a multinomial latent variable. Binary responses are linked via a probit link function. Figure 2 summarises the conditional probabilities that link these transitions for the model specification.

#### Model 3

We specified a third model, a latent disease profile model, that assumed an unconstrained transition matrix. This model was specified as a longitudinal probit regression model for the repeated dichotomous variable representing the answer to the question “Has the child had [a particular symptom] within the past 12 months?” where the symptoms were eczema, wheeze, and rhinitis. For each child, we assumed a uniform Dirichlet prior distribution with equal probability assignment to each latent class. The hyperparameter for the prior is a vector of pseudocounts or constants representing the “prior” number of observations in each of the *n* classes, and the posterior is estimated using expectation propagation. The tuning of hyperparameters is performed using posterior model parameters for each step of the inference algorithm using Bayesian inference. Model evidence (marginal likelihood) was tuned using posterior model parameters for each step of the inference algorithm derived by integrating out over all variables. We have specified a flat Gaussian prior on the variance, with a mean of zero and a precision of one.

**Figure 3:**
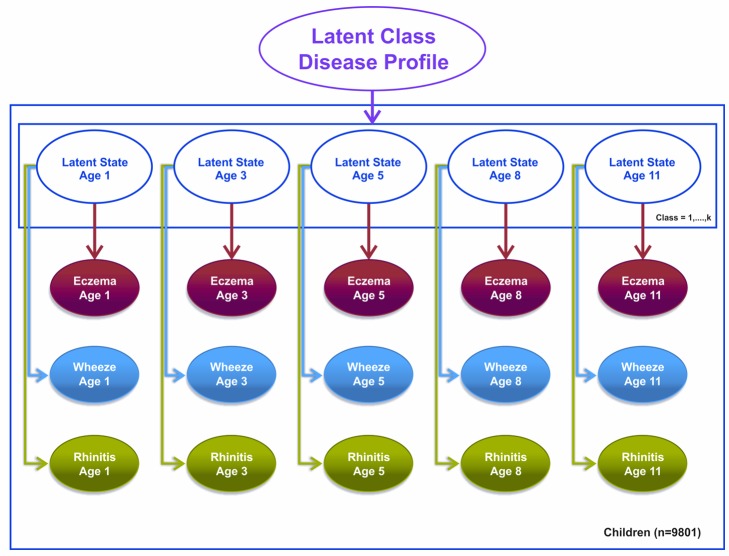
Graphical representation of the latent disease profile model taking into account the co-occurrence of symptoms at each time point (Model 3). Shaded circles represent observed variables, and unshaded circles represent latent variables to be inferred. Symptoms are joined together by a latent class disease profile.

This model assumes that other than random temporal fluctuation, each child's joint pattern of eczema, wheeze, and rhinitis can be explained by their belonging to a particular disease class profile. This model makes no constraints on (1) the structure of transitions within and between different symptoms over time or (2) the progression from one symptom to another. The conditional transition probabilities are evaluated and assumed to be independent. Figure 3 summarises this model.

Note that for these models, *t*+1 refers to the next time step at which data were collected. So, for example, if *t* is age 3 y, *t*+1 would be age 5 y. Likewise, t−1 refers to the previously observed time step. So, for example, if *t* is age 11 y, *t*−1 would be age 8 y.

Each of these three models was fitted on the full, combined dataset across both cohorts. In each of these three models, we assumed that each child belongs to one of *n* latent classes; the number and size of classes were not known a priori. Children belonging to the same class are similar with respect to the observed pattern of observations over time. These homogenous classes of children that we identified were assumed to come from the same probability distributions. For each child, we calculated the posterior probability of belonging to each of the latent classes, and assigned children to their highest probability class. We compared the models using model evidence, a measure of model goodness-of-fit, to identify the most parsimonious model [41].

In order to assess the possible effect of Lindley's paradox, which may occur when model evidence is mostly determined by the prior as opposed to the likelihood of the data, we specified a range of priors, setting the pseudocounts to 1/*n*, 2/*n*, 1, and 2.

Bayesian data analysis consists of three principle stages for statistical inference: first, specifying a prior or quantifying a hypothesis; second, assessing statistical modelling on the data that we have at hand; and third, updating our prior knowledge with the analysis of the data. For all three models, the uniform Dirichlet prior that we used allows us to assume that each child has an equal probability (1/*n*) of belonging to *n* classes, where we specified *n* to range from 2 to 15 classes. Using a Dirichlet prior is equivalent to an agnostic approach to our data analysis, where we assume that each child has an equal probability of belonging to each class and also that the number of children within each class is equal. For each child, we assumed a uniform Dirichlet prior distribution with equal probability assignment to each latent class. For the interested reader, the book *Bayesian Data Analysis* [42] by Gelman et al. provides insight on choosing uniform priors, and the book *Pattern Recognition and Machine Learning* [43] provides a more in-depth discussion of unsupervised learning methods such as the ones presented in our models.

We also investigated the longitudinal profiles using current asthma rather than wheeze.

In order to investigate whether similar profiles exist among children who may be classified as having moderate/severe eczema, in the MAAS cohort we carried out additional analysis excluding children with mild eczema. We defined mild eczema as parentally reported eczema, but no physician-prescribed topical corticosteroid.

Longitudinal analysis of the trajectories of atopic sensitisation in different latent classes in the MAAS population was performed using generalised estimating equations (Stata 12, StataCorp, College Station, Texas).

## Results

### Participant Flow

**Figure 4:**
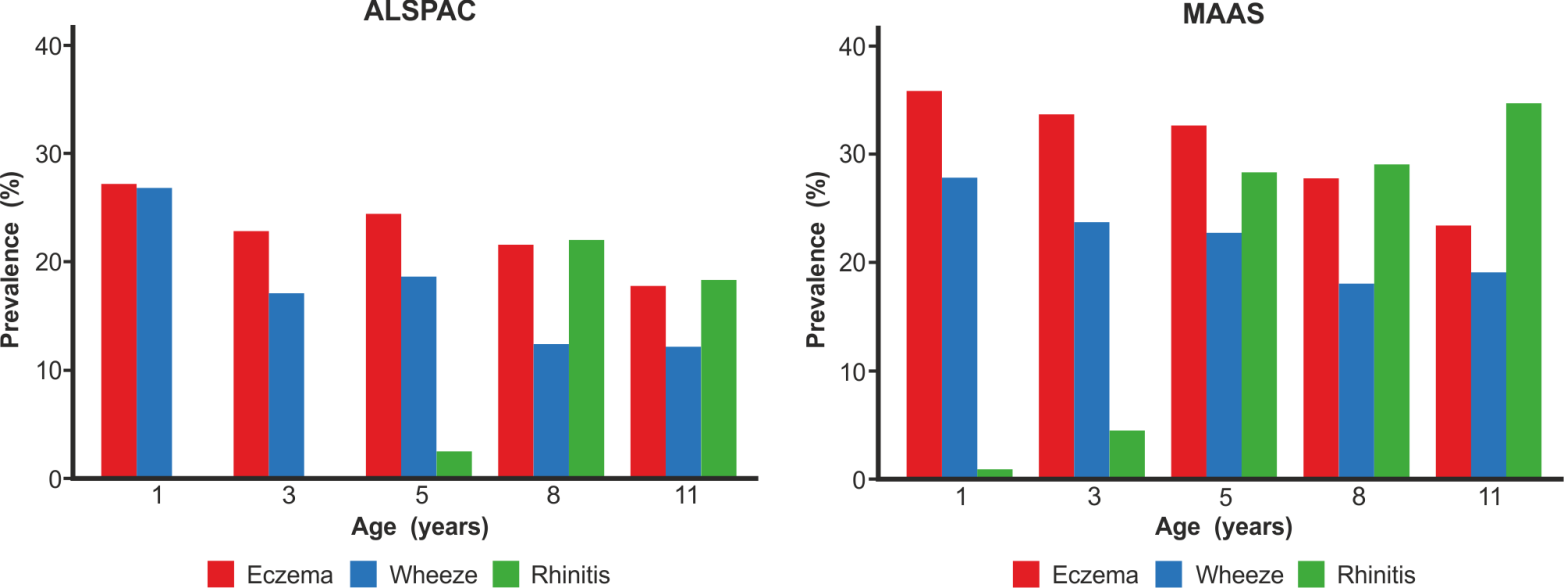
Prevalence of wheeze, eczema, and rhinitis over cross-sectional time points in the ALSPAC and MAAS cohorts.

We included a total of 9,801 children (8,665 from ALSPAC, 1,136 from MAAS). Characteristics of the study populations, including the prevalence of wheeze, eczema, and rhinitis at each time point, are presented in Table 1 and Figure 4. There were similar cross-sectional patterns across the two cohorts, with a steady decrease in wheeze and eczema with increasing age, whereas the prevalence of rhinitis increased with age.

**Table 1:** Characteristics of the two cohorts.

Characteristic	MAAS Cohort		ALSPAC Cohort		Joint MAAS and ALSPAC Cohort	
	*n*/Total	Percent	*n*/Total	Percent	*n*/Total	Percent
**Gender (Female)**	617/1,136	54.3	4,212/8,665	48.61	4,829/9,801	49.3
**Eczema**						
Age 1 y	383/1,077	35.6	2,263/8,328	27.2	2,646/9,405	28.1
Age 3 y	355/1,061	33.5	1,872/8,165	22.9	2,227/9,226	24.1
Age 5 y	340/1,050	32.4	1,974/8,080	24.4	2,314/9,130	25.3
Age 8 y	285/1,027	27.8	1,542/8,163	19.5	1,827/9,190	19.9
Age 11 y	216/924	23.4	1,252/7,047	17.8	1,468/7,971	18.4
**Wheeze**						
Age 1 y	300/1,087	27.6	2,210/8,276	26.7	2,510/9,363	26.8
Age 3 y	257/1,095	23.5	1,392/8,168	17.0	1,649/9,263	17.8
Age 5 y	238/1,056	22.5	1,496/8,130	18.4	1,734/9,186	18.9
Age 8 y	185/1,024	18.1	897/7,182	12.5	1,082/8,206	13.2
Age 11 y	173/916	18.9	841/6,958	12.1	1,014/7,874	12.9
**Rhinitis**						
Age 1 y	8/943	0.9			8/943	0.9
Age 3 y	49/1,075	4.6			49/1,075	4.6
Age 5 y	292/1,039	28.1	197/8,421	2.3	489/9,460	5.2
Age 8 y	297/1,027	28.9	1,438/6,513	22.1	1,128/7,540	23.0
Age 11 y	321/927	34.6	1,271/6,953	18.3	1,592/7,880	20.2
**Sensitisation**						
Age 1 y	55/484	11.4				
Age 3 y	222/970	22.9				
Age 5 y	289/952	30.4				
Age 8 y	314/927	33.9	874/5,624	15.5	1,188/6,551	18.1
Age 11 y	280/795	35.2				

### Latent Disease Profiles Identified

**Figure 5:**
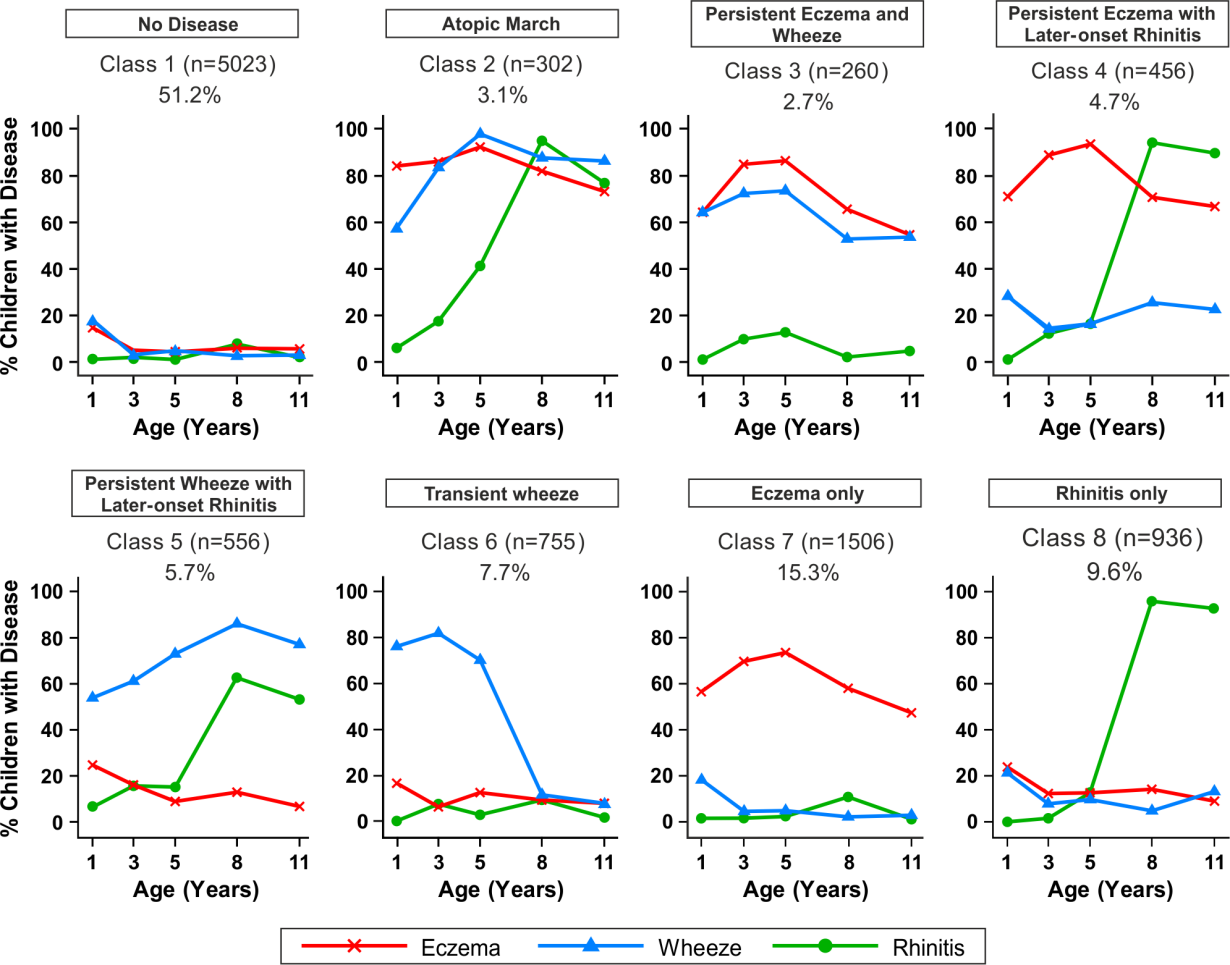
Distinct disease profile classes. Using Bayesian machine learning joint modelling of eczema, wheeze, and rhinitis across two population-based birth cohorts, we identified eight distinct disease profile classes. The number of children and the proportion of the study population are indicated for each class. Plots indicate longitudinal trajectories of wheeze, eczema, and rhinitis within each class.

Comparing model evidence, Model 1 (which assumed that disease transitions are independent) gave a better fit than Model 2 (which explicitly assumed atopic march transitions). However, these two models did not reach stable model convergence and were therefore not evaluated further. Stable model convergence was achieved in Model 3 (latent disease profile model); the optimal solution that best described the data was a model that inferred eight latent classes (Table S1). Posterior probabilities of class membership were generally high (Table S2). Based on our interpretation of their characteristics in relation to patterns of eczema, wheeze, and rhinitis during childhood (Figure 5), we assigned the following labels to the latent eight classes.

#### No disease (*n* = 5,023, 51.3%)

Children in this class had a low probability of eczema, wheeze, and rhinitis.

#### Atopic march (*n* = 302, 3.1%)

Children in this class had a high probability of eczema from infancy to age 11 y. The probability of wheeze increased with time. For rhinitis, the probability increased from zero at age 1 y to almost 100% by age 8 y. Eczema developed first, followed by wheeze, and then rhinitis; however, there was little evidence of resolution of eczema by age 11 y.

#### Persistent eczema and wheeze (*n* = 266, 2.7%)

There was similar probability of wheeze and eczema throughout childhood, likely as co-morbidities, with a low probability of rhinitis throughout childhood.

#### Persistent eczema with later-onset rhinitis (*n* = 456, 4.7%)

The prevalence of eczema increased steadily from ∼70% in early life to 95% at age 5 y, with little resolution to age 11 y. The probability of rhinitis increased to almost 100% by age 8 y. These children had a low probability of wheeze throughout childhood.

#### Persistent wheeze with later-onset rhinitis (*n* = 556, 5.7%)

This class was characterised by a high probability of wheeze throughout childhood, with increasing probability of rhinitis to almost 100% by age 11 y. Probability of eczema was low, declining steadily to age 11 y.

#### Transient wheeze (*n* = 755, 7.7%)

Children in this class had a high probability of wheeze within the first 5 y, with remission by age 8 y, and a very low probability of eczema and rhinitis throughout childhood.

#### Eczema only (*n* = 1,506, 15.3%)

High probability of eczema throughout life, peaking at ∼80% at age 5 y, then declining steadily to a 50% probability at age 11 y.

#### Rhinitis only (*n* = 936, 9.6%)

Children in this class had increasing probability of rhinitis from age 5 to 11 y, but no wheeze or eczema.

**Figure 6:**
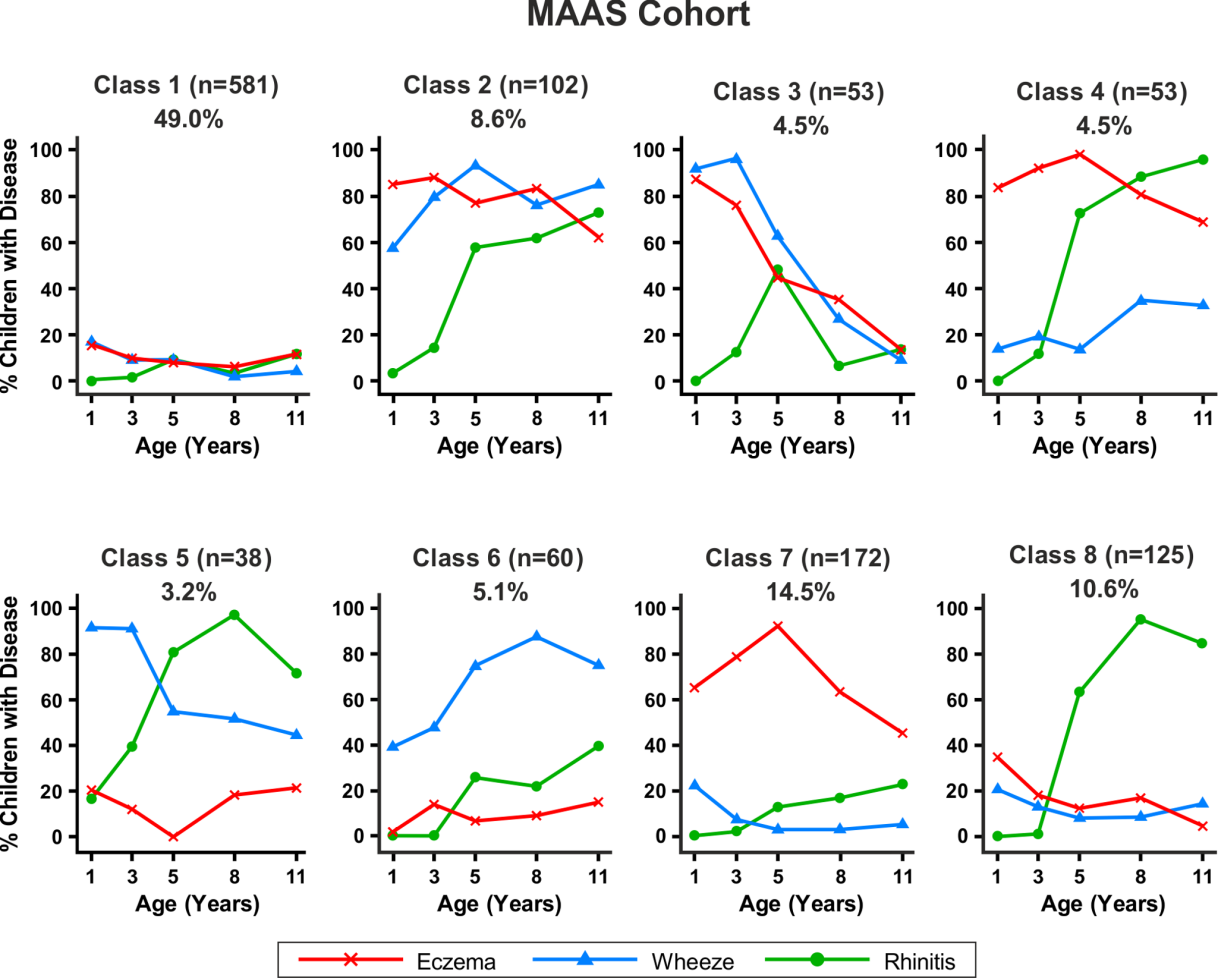
Distinct disease profile classes in MAAS. Bayesian machine learning joint modelling of eczema, wheeze, and rhinitis for the MAAS cohort. We identified eight distinct disease profile classes that best described the data. Class 1, no disease; class 2, atopic march; class 3, persistent eczema and wheeze; class 4, persistent eczema with later-onset rhinitis; class 5, persistent wheeze with later-onset rhinitis; class 6, transient wheeze; class 7, eczema only; class 8, rhinitis only.

**Figure 7:**
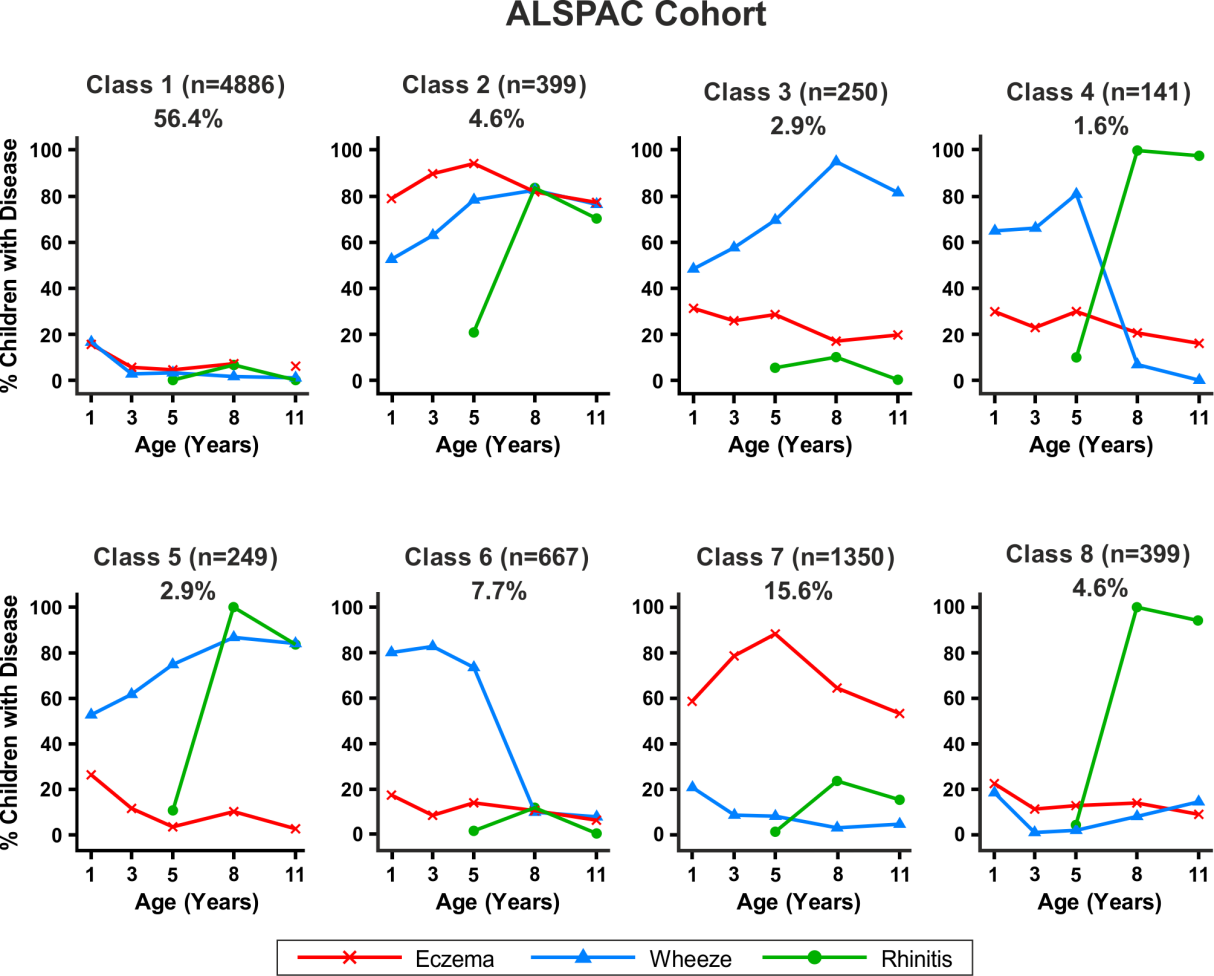
Distinct disease profile classes in ALSPAC. Bayesian machine learning joint modelling of eczema, wheeze, and rhinitis for the ALSPAC cohort. We identified eight distinct disease profile classes that best described the data. Class 1, no disease; class 2, atopic march; class 3, persistent eczema and wheeze; class 4, persistent eczema with later-onset rhinitis; class 5, persistent wheeze with later-onset rhinitis; class 6, transient wheeze; class 7, eczema only; class 8, rhinitis only.

Similar results were obtained when modelling was carried out separately for the MAAS and ALSPAC cohorts (Figures 6 and 7). Latent variable modelling using “current asthma” instead of “current wheeze” gave similar profiles (Figure S1).

Changing prior parameters by setting the pseudocounts to 1/*n*, 2/*n*, 1, and 2 gave consistent model evidence (Table S3) and results, identifying a small group of children who followed profiles similar to the atopic march.

### Latent Classes and Gender

There were no significant gender differences in the classes no disease, persistent eczema and wheeze, and persistent eczema with later-onset rhinitis (Table 2). The eczema only class contained a higher proportion of girls (57.4% versus 42.6%, *p*<0.001). In contrast, in all other classes there were more boys than girls (*p*<0.001), most notably in the atopic march (60.9% versus 39.1%) and persistent wheeze with later-onset rhinitis (62.8% versus 37.2%) classes.

**Table 2:** Gender distribution in different classes.

Class Number	Class	Female, *N* (Percent)	Male, *N* (Percent)	*p*-Value
1	No disease	2,456 (48.9)	2,567 (51.1)	Baseline
2	Atopic march	118 (39.1)	184 (60.9)	0.001
3	Persistent eczema and wheeze	128 (48.1)	138 (51.9)	0.81
4	Persistent eczema with later-onset rhinitis	223(48.9)	233 (51.1)	0.99
5	Persistent wheeze with later-onset rhinitis	207 (37.2)	349 (62.8)	<0.001
6	Transient wheeze	313 (41.5)	442 (58.5)	<0.001
7	Eczema only	864 (57.4)	642 (42.6)	<0.001
8	Rhinitis only	422 (45.1)	514 (54.9)	0.032
	**Total**	**4,731 (48.3)**	**5,070 (51.7)**	

### Trajectories of Sensitisation in Different Classes

**Figure 8:**
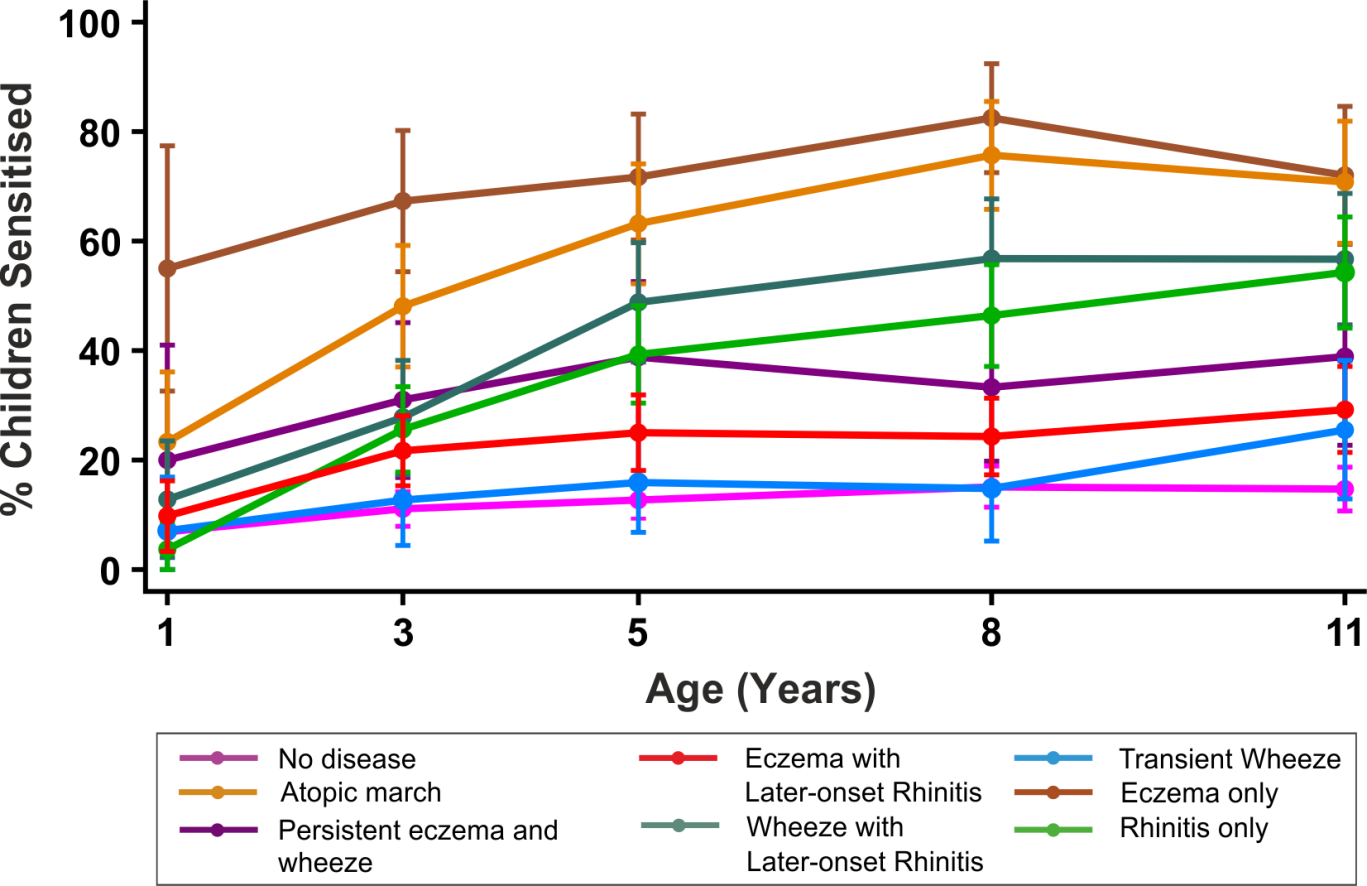
Proportion of children sensitised in each latent disease profile.

By age 11 y, 35.2% of children in MAAS were sensitised, increasing from 11.4% at age 1 y (Table 1). The trajectories of sensitisation in different classes are presented in Figure 8, and superimposed on different classes in Figure S2. For the classes persistent eczema and wheeze, persistent wheeze with later-onset rhinitis, and rhinitis only, the trajectories of sensitisation were very similar (*p*>0.2). Children in the rhinitis only class had a significantly higher risk of sensitisation than those in the eczema only class (odds ratio [OR] = 2.16, 95% confidence interval (CI) 1.48–3.16, *p*<0.001). There was no significant difference in the probability of sensitisation between children in the classes transient wheeze and no disease (*p* = 0.28), whilst children in all other classes were at an increased risk of sensitisation compared to the no disease class (Table S4). Children in the atopic march class were at the highest risk of sensitisation (OR = 21.92, 95% CI 13.56–35.42, *p*<0.001) (Table S4). Analysis using data on sensitisation from both MAAS and ALSPAC at age 8 y showed similar trends (Table 3), with children in the atopic march class having significantly higher risk of sensitisation than all other groups (*p*<0.001).

**Table 3:** Sensitisation distribution in different classes (data on sensitisation from both MAAS and ALSPAC cohorts at age 8 y).

Class Number	Class	Not Sensitised, *N* (Percent)	Sensitised, *N* (Percent)	OR (95% CI)	*p*-Value
1	No disease	3,036 (93.0)	228 (7.0)	Baseline	Baseline
2	Atopic march	62 (29.0)	152 (71.0)	32.6 (23.6–45.2)	<0.001
3	Persistent eczema and wheeze	136 (70.1)	58 (29.9)	5.7 (4.1–7.9)	<0.001
4	Persistent eczema with later-onset rhinitis	159 (47.5)	176 (52.5)	14.7 (11.4–19.0)	<0.001
5	Persistent wheeze with later-onset rhinitis	203 (53.8)	174 (46.2)	11.4 (9.0–14.6)	<0.001
6	Transient wheeze	439 (90.1)	48 (9.9)	1.5 (1.0–2.0)	0.024
7	Eczema only	869 (86.3)	138 (13.7)	2.1 (1.7–2.6)	<0.001
8	Rhinitis only	458 (68.2)	184 (31.8)	5.3 (4.3–6.7)	<0.001
	**Total**	**5,265 (81.9)**	**1,162 (18.1)**		

### Latent Disease Profiles among Children with Moderate/Severe Eczema

Within the MAAS cohort (for which we had relevant data to enable indirect ascertainment of eczema severity), we carried out further analyses excluding children with mild eczema to investigate disease profiles among children with moderate/severe eczema. We excluded data from 88 children with parentally reported eczema at age 1 or 3 y, but no general practitioner prescription for topical corticosteroids during this time (and therefore assumed to have mild eczema) and from 97 children with parentally reported eczema but missing general practitioner prescription data (so severity could not be confirmed). A total of 102 children had missing data on eczema at ages 1 and 3 y. We identified six classes. Based on our interpretation of their characteristics (Figure S3), we have labelled these classes as follows.

#### No disease (*n* = 473, 52.7%)

Children in this class had a low probability of eczema, wheeze, and rhinitis.

#### Atopic march (*n* = 63, 7.0%)

Children in this class had a high probability of eczema from infancy to age 11 y. The probability of wheeze increased with time. For rhinitis, the probability increased from 0% at age 1 to ∼80% by age 11 y. Eczema developed first, followed by wheeze, and then rhinitis; however, there was little evidence of resolution of eczema by age 11 y.

#### Transient early eczema and wheeze (*n* = 71, 7.9%)

Eczema was present at age 1 and 3 y, and then the probability of eczema diminished at age 5 y, with no eczema by age 11 y. These children also had a decreasing probability of wheeze over time, with little rhinitis.

#### Wheeze/wheeze with later-onset rhinitis (*n* = 92, 10.3%)

Children in this class had a high probability of wheeze throughout childhood, with a ∼40% probability of rhinitis by age 5 y.

#### Eczema/eczema with later-onset rhinitis (*n* = 94, 10.3%)

Children in this class had a high probability of eczema throughout childhood, with a ∼60% probability of rhinitis by age 11 y.

#### Rhinitis only (*n* = 104, 11.6%)

Children in this class had increasing probability of rhinitis from age 5 to 11 y, but no wheeze or eczema.

These classes were strongly associated with those identified using joint modelling of all data, including children with mild eczema (*p*<0.0001; Table S5). The exception was a new class of children with transient early eczema and no other symptoms. Of note, a similar class was identified when we analysed data from the MAAS cohort only (Figure 6, class persistent eczema and wheeze). Of note, in the model excluding children with mild eczema, 46.1% of children developed wheeze or rhinitis independent of eczema (classes wheeze/wheeze with later-onset rhinitis and rhinitis only). This proportion is similar to the results of the model including all children with eczema, in which 45.6% of children developed wheeze or rhinitis independent of eczema.

We note that because of the smaller number of children, the model excluding children with mild eczema is poorer at distinguishing (1) between children with wheeze with later-onset rhinitis and children with wheeze only (57.6% of these children were identified as wheeze with later-onset rhinitis and 40.2% were identified as wheeze only in the original model) and (2) between children with eczema only and children with eczema with later-onset rhinitis (55.3% of these children were identified as eczema with later-onset rhinitis and 40.4% as eczema only in the original model).

The data suggest that including children with mild eczema resulted in a greater certainty in class assignment.

## Discussion

By jointly modelling longitudinal data from two independent population-based birth cohorts, we have identified eight different classes describing the temporal trajectories of eczema, wheeze, and rhinitis during childhood. Less than 7% of children with symptoms followed a trajectory profile resembling the atopic march, in which early eczema and wheeze were followed by rhinitis. In contrast to the commonly held view [2],[6],[7], we saw little evidence of resolution of eczema in this class. The remaining six disease classes were generally characterised by the presence of only one or two of the three symptoms, indicating that more than 90% of children with symptoms commonly associated with atopy in childhood do not follow the widely perceived trajectory of the atopic march. Furthermore, highly concordant patterns of sensitisation over time were associated with very different profiles of symptoms; for example, the classes persistent eczema and wheeze, persistent wheeze with later-onset rhinitis, and rhinitis only had similar temporal patterns of sensitisation. Conversely, for the three classes characterised by persistence of wheeze—atopic march, persistent eczema, and wheeze, persistent wheeze with later-onset rhinitis—patterns of sensitisation were different, as were patterns of co-morbid eczema and rhinitis. This may suggest uncoupling of the mechanisms leading to atopic sensitisation and those leading to symptoms, and is consistent with the recent discovery of different phenotypes of atopy characterised by different patterns of IgE responses to allergens (both over time and to different individual allergens) that differ in their associations with clinical symptoms [29],[30],[44]. Future work may be carried out to investigate whether there are underlying biological and genetic markers associated with these potentially distinct mechanisms.

### Limitations and Strengths

The questions that we used in the two cohorts contained small differences in the wording (e.g., in MAAS, the wheezing question asked about “wheezing or wheezing with whistling in the chest”, whereas in ALSPAC, the question asked about “wheezing with whistling on his/her chest when he/she breathed”). However, these differences were small, and it is unlikely that they have affected the results.

The differences in the prevalence of rhinitis between the two cohorts may be of concern. However, it is striking that when we modelled the data separately for each cohort, although the prevalence of rhinitis was higher in MAAS compared to ALSPAC, the results indicated similar latent profiles, suggesting consistent patterns of symptoms across the two populations.

Data on confirmed food allergy were not available from early life in either cohort, and was therefore not included in the model. Sensitisation data were available for ALSPAC at only one time point (age 8 y); we therefore used data only from MAAS to compare longitudinal patterns of sensitisation across the latent classes, and the analysis of probabilities of sensitisation across the two cohorts was limited to age 8 y.

We acknowledge that the definition of eczema that we used in our models is based on parental reporting using validated questionnaires (as in most other epidemiological studies), and that this may lead to overestimation of the true prevalence. For the MAAS cohort, we extracted all data from the primary care medical records, which enabled comparison between physician-diagnosed and parentally reported eczema. For ∼85% of children whose parents reported eczema, eczema was confirmed in the primary care records, indirectly suggesting that overestimation of the prevalence of eczema is unlikely.

One of the major strengths of this study is the ability to disambiguate the temporal structure of disease profiles, thus allowing us to identify groups of children with similar patterns of the onset and progression of eczema, wheeze, and rhinitis over time. It is striking that when we modelled the data separately for each cohort, although there was a higher prevalence of rhinitis in MAAS compared to ALSPAC, the results indicated similar latent profiles, suggesting consistent evidence across the two populations. The joint modelling of both MAAS and ALSPAC data gave stronger evidence for these patterns. A further advantage of joint modelling is the ability to borrow strength using complex machine learning models, and obtain better statistical models to predict expected profiles based on the presence of symptoms across the two cohorts. For example, this approach enabled learning about early-life rhinitis parameters in ALSPAC (where these data were absent) from the MAAS data. Similarly, the larger sample size in ALSPAC, when combined with MAAS, gave a greater resolution in identifying patterns of disease profiles and assigning children to different classes with less uncertainty.

### Interpretation

**Figure 9:**
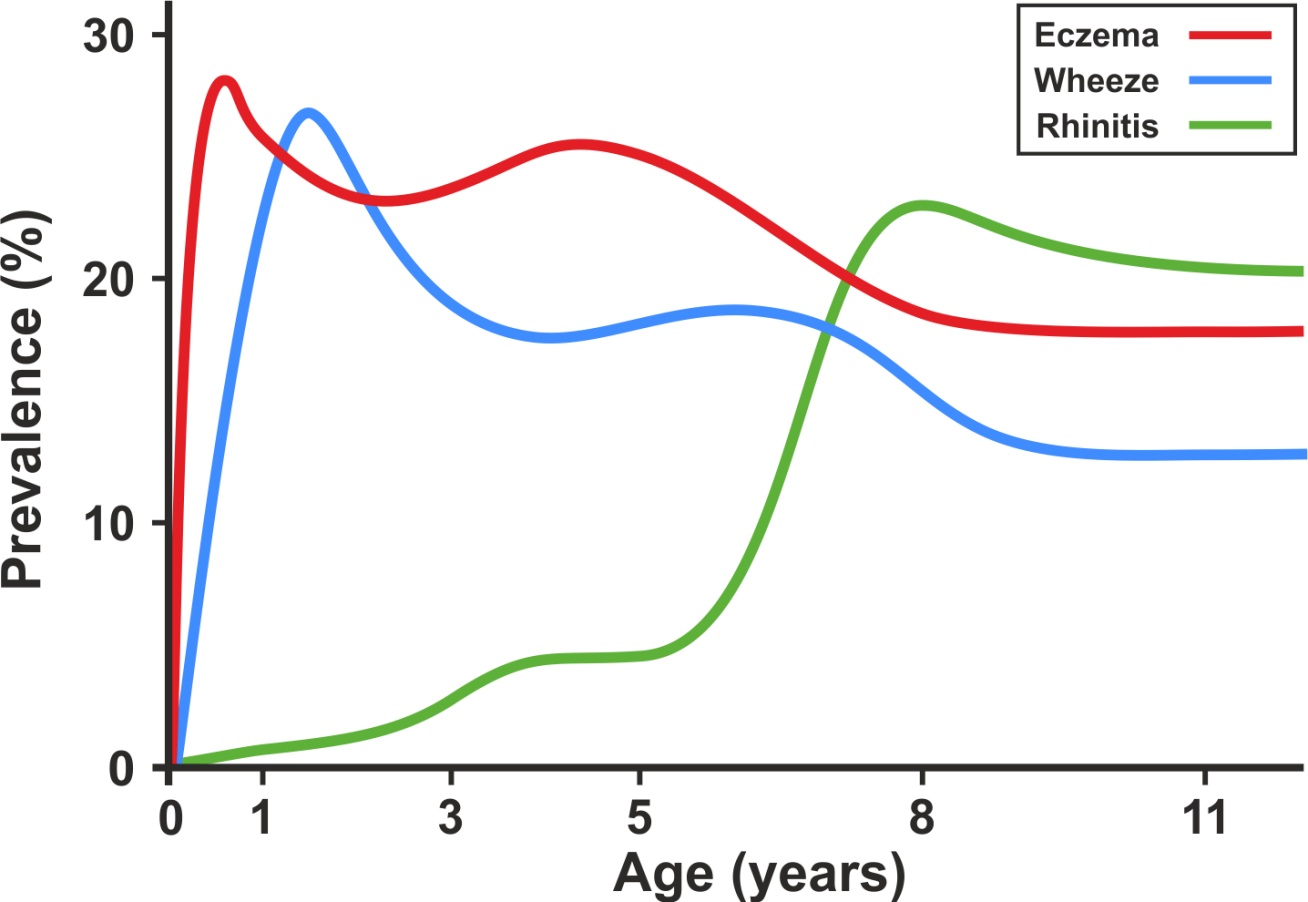
Profile plot showing cross-sectional change in prevalence of eczema, wheeze, and rhinitis in the ALSPAC and MAAS birth cohorts.

When we present the prevalence data from each time point cross-sectionally in our two cohorts (Figure 9), the profile appears strikingly similar to the traditional concept of atopic march [7]. It is clear that this traditional approach reflects patterns at the population level, rather than the natural covariance of symptoms within individuals' life courses. We have applied computationally intensive techniques of machine learning coupled with Bayesian inference to model the data longitudinally, thereby allowing the identification of a latent structure within the data that may better reflect the individual life course (as opposed to changes in prevalence at the population level). These approaches allowed us to construct customised models for longitudinal data in order to explicitly incorporate uncertainty and investigate hypotheses regarding the co-occurrence of eczema, wheeze, and rhinitis over time. By explicitly modelling the assumptions underlying the atopic march (that the profile of this march of symptoms may follow a chronological manifestation of eczema, wheeze, and then rhinitis), we found that this model does not capture patterns in the data as well as a model in which we assumed that eczema, wheeze, and rhinitis follow independent trajectories. A recent review has challenged the lack of robust evidence for inferring that eczema is causally linked to asthma and rhinitis [4]. Our results suggest that the relationship between eczema, wheeze, and rhinitis is not causal, but can be explained by an underlying latent (non-observed) disease status within the population, whose structure we were able to infer by investigating individual longitudinal disease profiles. We propose that eczema, wheeze, and rhinitis are all common and so co-exist, but mostly as independent entities, each associated to a greater or lesser degree with atopic sensitisation. Our results show that although there is a group of children with trajectories similar to the atopic march paradigm, this group comprises approximately one child in every 20 with symptoms. Of note, a striking characteristic of this class is a very strong association with atopy.

A recent study provided evidence in support of atopic march in relation to early-life mild eczema in the general population [3]. We acknowledge that atopic march may be associated with severe/moderate eczema, and that the likely inclusion of very mild eczema in our analysis may have skewed the results. To address this concern, within the MAAS cohort (in which we have relevant data to indirectly ascertain eczema severity), we carried out further analyses excluding children with mild eczema to investigate disease profiles among children with moderate/severe eczema (and those without eczema). We identified similar classes with profiles similar to those identified when using joint modelling of all the data. It is of note that even when children with mild eczema were excluded from the analysis, the atopic march class accounted for only 5.8% of the general population.

Four of the classes included eczema as a characteristic feature (atopic march, persistent eczema and wheeze, eczema with later-onset rhinitis, and eczema only). It is important to note that by far the largest of these was the eczema only class (15.4% of the total population, including 60% of children within classes in which eczema was a characteristic feature). In this class (uniquely characterised by female predominance), eczema occurred generally without wheeze or rhinitis, suggesting that most children with early eczema continue having eczema only, and do not progress to develop co-morbidities. In the persistent eczema and wheeze class, eczema and wheeze occurred as co-morbidities (i.e., wheeze did not follow after eczema). Only in the atopic march class (accounting for only 12% of children assigned to a class where eczema was a characteristic feature) was there any suggestion that eczema may precede wheeze. This would argue against the relevance of the atopic march for most children with eczema. Thus, using eczema as the indicator of subsequent risk of asthma, and assigning “preventative” measures to this group [13], is flawed, given that most children with early eczema will not develop asthma. The results of our study emphasise the need for clinicians to acknowledge the heterogeneity of patterns of “allergic” diseases and to communicate to parents the uncertainty inherent in predicting the development of new symptoms or resolution of existing ones.

### Conclusions

Our results provide evidence that suggests that the developmental profiles of eczema, wheeze, and rhinitis are heterogeneous, and that only a small proportion of children follow a trajectory profile similar to that of the atopic march.

## Supporting Information

Figure S1 **Bayesian machine learning joint modelling of eczema, asthma, and rhinitis for both the ALSPAC and MAAS cohorts.** We identified eight distinct disease profile classes that best described the data.
(DOCX)

Figure S2 **Profiles of sensitisation in the MAAS cohort across latent classes identified via jointly modelling data from both the ALSPAC and MAAS cohorts.**
(DOCX)

Figure S3 **Bayesian machine learning joint modelling of eczema, wheeze, and rhinitis for the MAAS cohort, excluding children with mild eczema.**
(DOCX)

Table S1 **Model evidence for different numbers of inferred latent classes.** Evidence was assessed on 100 random samples of hold-out data. Higher evidence indicates better model fit. The optimal solution was a model that inferred eight latent disease profiles. Model convergence was assumed when model evidence remained stable for 100 consecutive iterations.
(DOCX)

Table S2 **Posterior probabilities of class membership.** This is the conditional probability that a child is assigned to a particular (predicted) class given their actual assigned class membership.
(DOCX)

Table S3 **Model evidence for different numbers of inferred latent classes with different priors.** This table compares priors where the number of pseudocounts is set to from 1/*n* up to 2 to investigate whether setting different priors influences model evidence.
(DOCX)

Table S4 **Odds ratios with 95% confidence intervals for the association of latent disease profile classes with longitudinal sensitisation in the MAAS cohort. Models were adjusted for age.**
(DOCX)

Table S5 **Confusion matrix showing association between classes obtained excluding and including children with mild eczema in the MAAS cohort.**
(DOCX)

## Abbreviations

ALSPAC: Avon Longitudinal Study of Parents and Children
CI: confidence interval
ISAAC: International Study of Asthma and Allergies in Childhood
MAAS: Manchester Asthma and Allergy Study
OR: odds ratio
