# Supplementary material for: Developmental Profiles of Eczema, Wheeze, and Rhinitis: Two Population-Based Birth Cohort Studies
Source: PLoS Med. 2014 Oct 21;11(10):e1001748. doi: 10.1371/journal.pmed.1001748 (PMC4204810; doi:10.1371/journal.pmed.1001748)
Supplement: Figure S1 — Bayesian machine learning joint modelling of eczema, asthma, and rhinitis for both the ALSPAC and MAAS cohorts. We identified eight distinct disease profile classes that best described the data. (DOCX) [file pmed.1001748.s001.docx]

**Supplementary Figure S1: Bayesian machine learning joint modelling of eczema, asthma, and rhinitis for both the ALSPAC and MAAS cohorts.**

**We identified 8 distinct disease profile classes that best described the data.**

**Order of the classes show similarity with the joint modelling of eczema, wheeze and rhinitis.**
