# Supplementary material for: Developmental Profiles of Eczema, Wheeze, and Rhinitis: Two Population-Based Birth Cohort Studies
Source: PLoS Med. 2014 Oct 21;11(10):e1001748. doi: 10.1371/journal.pmed.1001748 (PMC4204810; doi:10.1371/journal.pmed.1001748)
Supplement: Figure S2 — Profiles of sensitisation in the MAAS cohort across latent classes identified via jointly modelling data from both the ALSPAC and MAAS cohorts. (DOCX) [file pmed.1001748.s002.docx]

**Supplementary Figure S2: Profiles of sensitisation in the MAAS cohort across latent classes identified via jointly modelling data from both the ALSPAC and MAAS cohorts.**
