# Supplementary material for: Developmental Profiles of Eczema, Wheeze, and Rhinitis: Two Population-Based Birth Cohort Studies
Source: PLoS Med. 2014 Oct 21;11(10):e1001748. doi: 10.1371/journal.pmed.1001748 (PMC4204810; doi:10.1371/journal.pmed.1001748)
Supplement: Figure S3 — Bayesian machine learning joint modelling of eczema, wheeze, and rhinitis for the MAAS cohort, excluding children with mild eczema. (DOCX) [file pmed.1001748.s003.docx]

**Supplementary Figure S3: Bayesian machine learning joint modelling of eczema, wheeze, and rhinitis for the MAAS cohort, excluding children with mild eczema.**
