# Supplementary material for: Developmental Profiles of Eczema, Wheeze, and Rhinitis: Two Population-Based Birth Cohort Studies
Source: PLoS Med. 2014 Oct 21;11(10):e1001748. doi: 10.1371/journal.pmed.1001748 (PMC4204810; doi:10.1371/journal.pmed.1001748)
Supplement: Table S1 — Model evidence for different numbers of inferred latent classes. Evidence was assessed on 100 random samples of hold-out data. Higher evidence indicates better model fit. The optimal solution was a model that inferred eight latent disease profiles. Model convergence was assumed when model evidence remained stable for 100 consecutive iterations. (DOCX) [file pmed.1001748.s004.docx]

**Supplementary Table S1:** Model evidence for different numbers of inferred latent classes.

Evidence was assessed on 100 random samples of hold-out data. Higher evidence indicates better model fit. The optimal solution was a model which that inferred eight latent disease profiles. Model convergence was assumed when model evidence remained stable for 100 consecutive iterations.

|  | Table of Model Evidence | | | | | | | | |
| --- | --- | --- | --- | --- | --- | --- | --- | --- | --- |
|  | **Number of Inferred Classes** | | | | | | | | |
|  | | **2** | **3** | **4** | **5** | **6** | **7** | **8** | **9** |
| ALSPAC | | -42160 | -40699 | -40074 | -39422 | -39323 | **-39153** | -39183 | -39895 |
| MAAS | | -7455 | -7321 | -7269 | -7185 | -7103 | -7096 | **-7094** | -7454 |
| Joint ALSPAC + MAAS | | -49920 | -48448 | -47506 | -46930 | -46845 | -46658 | **-46503** | 46424* |

*Model convergence not achieved
