# Supplementary material for: Developmental Profiles of Eczema, Wheeze, and Rhinitis: Two Population-Based Birth Cohort Studies
Source: PLoS Med. 2014 Oct 21;11(10):e1001748. doi: 10.1371/journal.pmed.1001748 (PMC4204810; doi:10.1371/journal.pmed.1001748)
Supplement: Table S2 — Posterior probabilities of class membership. This is the conditional probability that a child is assigned to a particular (predicted) class given their actual assigned class membership. (DOCX) [file pmed.1001748.s005.docx]

**Supplementary Table S2:** Posterior probabilities of class membership. This is the conditional probability that a child is assigned to a particular (predicted) class given their actual assigned class membership.

|  | Class | | | | | | | |
| --- | --- | --- | --- | --- | --- | --- | --- | --- |
| 1 | **2** | **3** | **4** | **5** | **6** | **7** | **8** |  |
| 0.943 | 0.924 | 0.783 | 0.805 | 0.805 | 0.756 | 0.805 | 0.846 |  |
