# Supplementary material for: Developmental Profiles of Eczema, Wheeze, and Rhinitis: Two Population-Based Birth Cohort Studies
Source: PLoS Med. 2014 Oct 21;11(10):e1001748. doi: 10.1371/journal.pmed.1001748 (PMC4204810; doi:10.1371/journal.pmed.1001748)
Supplement: Table S3 — Model evidence for different numbers of inferred latent classes with different priors. This table compares priors where the number of pseudocounts is set to from 1/n up to 2 to investigate whether setting different priors influences model evidence. (DOCX) [file pmed.1001748.s006.docx]

**Supplementary Table S3:** Model evidence for different numbers of inferred latent classes with different priors.

This table compares priors where the number of pseudo-counts is set to from 1/n up to 2 to investigate whether setting different priors influences model evidence.

|  | Table of Model Evidence | | | | | | | |
| --- | --- | --- | --- | --- | --- | --- | --- | --- |
|  | **Number of Inferred Classes** | | | | | | | |
| Prior on the number of pseudo-counts | **2** | **3** | **4** | **5** | **6** | **7** | **8** | **9** |
| 1/n | -50177 | -49030 | -48297 | -47774 | -47367 | -47130 | **-46989** | -47109* |
| 2/n | -50200 | -49104 | -48310 | -47797 | -47357 | -47143 | **-46994** | -47334* |
| 1 | -49920 | -48448 | -47506 | -46930 | -46845 | -46658 | **-46503** | -46424* |
| 2 | -49920 | -48448 | -47506 | -46930 | -46845 | -46733 | **-46596** | -46431* |
