# Supplementary material for: Developmental Profiles of Eczema, Wheeze, and Rhinitis: Two Population-Based Birth Cohort Studies
Source: PLoS Med. 2014 Oct 21;11(10):e1001748. doi: 10.1371/journal.pmed.1001748 (PMC4204810; doi:10.1371/journal.pmed.1001748)
Supplement: Table S4 — Odds ratios with 95% confidence intervals for the association of latent disease profile classes with longitudinal sensitisation in the MAAS cohort. Models were adjusted for age. (DOCX) [file pmed.1001748.s007.docx]

**Supplementary Table S4: Odds ratios (OR) with 95% confidence intervals for the association of latent disease profile classes with longitudinal sensitisation in the MAAS cohort. Models were adjusted for age.**

|  | |  | OR (95% CI) | p-value |
| --- | --- | --- | --- | --- |
| 1. | **No Disease** | | (baseline) |  |
| 2. | **Atopic March** | | 21.92 (13.56 - 35.42) | <0·001 |
| 3. | **Persistent Eczema and Wheeze** | | 3.67 (2.23 - 6.06) | <0·001 |
| 4. | **Persistent Eczema with Later-onset Rhinitis** | | 11.02 (7.34 - 16.52) | <0·001 |
| 5. | **Late-onset Wheeze and Rhinitis** | | 6.08 (4.11 - 8.99) | <0·001 |
| 6. | **Transient-Early Wheeze** | | 1.35 (0.79 - 2.31) | 0.277 |
| 7. | **Eczema only** | | 2.18 (1.54 - 3.09) | <0·001 |
| 8. | **Rhinitis only** | | 4.66 (3.27 - 6.63) | <0·001 |
