# Supplementary material for: Developmental Profiles of Eczema, Wheeze, and Rhinitis: Two Population-Based Birth Cohort Studies
Source: PLoS Med. 2014 Oct 21;11(10):e1001748. doi: 10.1371/journal.pmed.1001748 (PMC4204810; doi:10.1371/journal.pmed.1001748)
Supplement: Table S5 — Confusion matrix showing association between classes obtained excluding and including children with mild eczema in the MAAS cohort. (DOCX) [file pmed.1001748.s008.docx]

|  | Classes excluding Children with Mild Eczema | | | | | |
| --- | --- | --- | --- | --- | --- | --- |
| Original Classes from Joint Model | **No Disease** | **Atopic March** | **Transient Eczema** | **Wheeze only** | **Eczema only** | **Rhinitis only** |
|  | *n (%)* | *n (%)* | *n (%)* | *n (%)* | *n (%)* | *n (%)* |
| No Disease | 389 (82.2) | 0 | 0 | 0 | 0 | 0 |
| Atopic March | 0 | 38 (60.3) | 0 | 1 (1.1) | 0 | 0 |
| Persistent Eczema and Wheeze | 0 | 15 (23.8) | 14 (19.7) | 2 (2.2) | 3 (3.2) | 0 |
| Persistent Eczema with Later-onset Rhinitis | 0 | 4 (6.4) | 5 (7.0) | 0 | 51 (54.3) | 1 (1.0) |
| Persistent Wheeze with Later-onset Rhinitis | 0 | 6 (9.5) | 5 (7.0) | 57 (62.0) | 0 | 2 (1.9) |
| Wheeze only | 27 (5.7) | 0 | 3 (4.2) | 32 (34.8) | 0 | 0 |
| Eczema only | 44 (9.3) | 0 | 40 (56.3) | 0 | 39 (41.5) | 0 |
| Rhinitis only | 13 (2.8) | 0 | 4 (5.6) | 0 | 1 (1.1) | 101 (97.1) |
| *Total* | *473 (52.7)* | *63 (7.0)* | *71 (7.9)* | *92 (10.3)* | *94 (10.5)* | *104 (11.6)* |

**Supplementary Table S5:** Confusion Matrix showing association between classes obtained excluding and including children with mild eczema in the MAAS cohort
